# Alterations of Nuclear Architecture and Epigenetic Signatures during African Swine Fever Virus Infection

**DOI:** 10.3390/v7092858

**Published:** 2015-09-15

**Authors:** Margarida Simões, José Rino, Inês Pinheiro, Carlos Martins, Fernando Ferreira

**Affiliations:** 1 CIISA, Faculdade de Medicina Veterinária, Universidade de Lisboa, Avenida Universidade Técnica, 1300-477 Lisboa, Portugal; maggiesimo@gmail.com (M.S.); cmartins@fmv.ulisboa.pt (C.M.); 2 Instituto de Medicina Molecular, Faculdade de Medicina, Universidade de Lisboa, 1649-028 Lisboa, Portugal; joserino@medicina.ulisboa.pt; 3 Department of Epigenetics, Max Planck Institute of Immunobiology and Epigenetics, 79108 Freiburg, Germany; inespirespinheiro@gmail.com

**Keywords:** African swine fever virus, nuclear speckles, Cajal bodies, promyelocytic leukaemia nuclear bodies, heterochromatin protein 1, histone H3 methylation modifications, histone deacetylases

## Abstract

Viral interactions with host nucleus have been thoroughly studied, clarifying molecular mechanisms and providing new antiviral targets. Considering that African swine fever virus (ASFV) intranuclear phase of infection is poorly understood, viral interplay with subnuclear domains and chromatin architecture were addressed. Nuclear speckles, Cajal bodies, and promyelocytic leukaemia nuclear bodies (PML-NBs) were evaluated by immunofluorescence microscopy and Western blot. Further, efficient PML protein knockdown by shRNA lentiviral transduction was used to determine PML-NBs relevance during infection. Nuclear distribution of different histone H3 methylation marks at lysine’s 9, 27 and 36, heterochromatin protein 1 isoforms (HP1α, HPβ and HPγ) and several histone deacetylases (HDACs) were also evaluated to assess chromatin status of the host. Our results reveal morphological disruption of all studied subnuclear domains and severe reduction of viral progeny in PML-knockdown cells. ASFV promotes H3K9me3 and HP1β foci formation from early infection, followed by HP1α and HDAC2 nuclear enrichment, suggesting heterochromatinization of host genome. Finally, closeness between DNA damage response factors, disrupted PML-NBs, and virus-induced heterochromatic regions were identified. In sum, our results demonstrate that ASFV orchestrates spatio-temporal nuclear rearrangements, changing subnuclear domains, relocating Ataxia Telangiectasia Mutated Rad-3 related (ATR)-related factors and promoting heterochromatinization, probably controlling transcription, repressing host gene expression, and favouring viral replication.

## 1. Introduction

African swine fever virus (ASFV) causes a devastating disease of domestic pigs that is endemic in most of the African sub-Saharan region and has recently been identified in the Trans-Caucasian countries, Russian Federation [1] and in EU countries namely Poland, Lithuania, Latvia, and Estonia [2]. This disease poses a threat to global swine industry due to its potential dissemination by different means, further complicated by the lack of either an efficient treatment or vaccine. ASFV is a large dsDNA virus (170–190 kb) and the sole member of *Asfarviridae*, recently classified into the Nucleocytoplasmic Large DNA Viruses superfamily [3]. Although some studies report interactions between this virus and nuclear transcription factors [4], nuclear lamina disassembly [5], and DNA damage response activation [6,7], little is known about the impact of ASFV infection on the nuclear architecture. Further, we have recently demonstrated that ASFV replicates its genome inside the host nucleus [8], suggesting that ASFV infection could be abrogated by interfering with host nuclear factors and mechanisms. Research on nuclear spatial organization has unravelled strategies that viruses coevolved to exploit host cell nucleus [9,10]. Some viruses induce morphological and functional changes in subnuclear domains and/or modify the epigenetically determined chromatin state of the host to accomplish successful infections [11,12]. As a result of the host transcription switch and downregulation of splicing events, nuclear speckles become unusually enlarged during viral infections due to the accumulation of small nuclear ribonucleoproteins (snRNPs) [13,14,15]. Viruses can also reshape Cajal bodies (CBs) by inducing accumulation of snRNPs and inhibiting RNA processing [16,17,18]. Likewise, promyelocytic leukaemia nuclear bodies (PML-NBs) are frequently disrupted by viruses for their involvement in antiviral responses and as viral replication centres preferentially associate to these domains (reviewed in [11,19]). In addition, PML-NBs have been implicated in DNA damage sensing [20] and showed to either exert antiviral responses or to facilitate viral infections [19,21,22].

Upon cell entry, several viruses elicit an epigenetic reprogramming of the host chromatin by changing the histone methylation status and subverting chromatin-associated enzymes, ultimately modifying chromatin structure and gene expression [23,24]. In eukaryotic cells, euchromatin is an open-packed form of DNA, transcriptionally active, and easily accessed for replication, while heterochromatin is constituted by genomic regions that are tightly packed and silenced [25,26]. Recently, it has been shown that heterochromatin formation is dependent on the enrichment of specific histone H3 methylation marks, which, in turn, can recruit heterochromatin protein 1 isoforms (HP1α and HP1β), facilitating a repressive chromatin environment and DNA damage response [27,28,29,30,31]. Some viruses can also modify the methylation patterns of histone H3, hence regulating the accessibility of replication/transcription machineries to host chromatin domains and territories [32]. In addition, and similarly, histone deacetylases (HDACs) that are involved in heterochromatin formation and spreading, can display corrupted activities during infections to aid virus replication [33,34].

The aim of our study was to characterize the effect of ASFV infection on nuclear speckles, Cajal bodies, PML-NBs and, on host chromatin structure by using the well-established *in vitro* model (Vero cells infected with ASFV Ba71V isolate). Clear morphological changes (shape, size and number) were identified in all subnuclear domains. Additionally, PML-NBs showed a tendency to become juxtaposed to the activated DNA damage response (DDR) factors foci and further investigation disclosed a proviral role for PML protein in ASFV infection, since viral progeny was decreased in PML-knockdown cells. From early time points post-infection, ASFV also promotes the heterochromatinization of the host nucleus by inducing hypermethylation of specific lysine residues of histone H3 (H3K9me3 and H3K27me3), foci formation of the HP1β isoform and redistribution of activated DDR factors (p-p53 and pATR). Later, ASFV-infected cells showed HP1α and HDAC2 redistributed patterns suggesting that the heterochromatic state of the host chromatin is tightly controlled by the virus. Reinforcing this transcriptional repressive environment of the host, all studied subnuclear domains displayed a close proximity to heterochromatic regions in ASFV-infected cells.

In summary, our findings demonstrate that ASFV alters nuclear architecture by disrupting subnuclear domains and chromatin texture, whereas PML protein has a proviral role. The novel virus-host interactions here described uncover promising molecular targets to be tested for antiviral therapies (e.g., PML, HP1, and HDAC I inhibitors). Correspondingly, future research on ASFV proteins involved in the modulation of nuclear architecture and chromatin structure might open new insights for a more rational design of viral mutants to be used as efficient vaccines.

## 2. Results

### 2.1. ASFV Disrupts Host Subnuclear Domains

**Figure 1:**
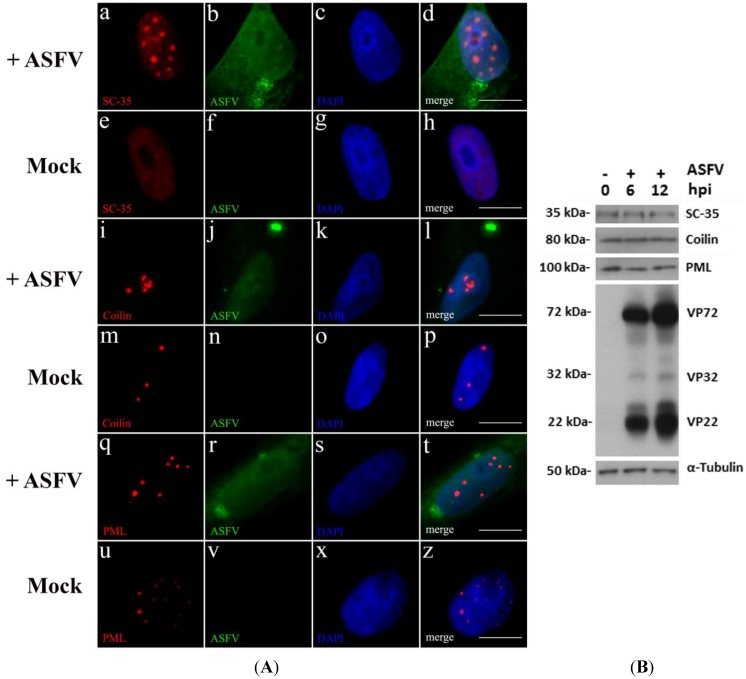
(**A**) ASFV induces the reorganization of subnuclear domains. Vero cells were infected with ASFV Ba71V isolate (MOI of 5), fixed at 6 hpi, permeabilized and immunostained; cell nuclei stained with DAPI (blue). (**a**–**d**) ASFV-infected cells (green) reveal globular and enlarged accumulations of SC-35 (red), while in non-infected cells (**e**–**h**), nuclear speckles (SC-35) show a pan-nuclear staining. (**i**–**l**) In ASFV-infected Vero cells (green), Cajal bodies (coilin, red) display “comma-shaped” morphology and group together, contrasting with the few pin-point bright foci of non-infected cells (**m**–**p**). (**q**–**t**) PML-NBs (PML, red) of infected cells (green) reveal fewer and enlarged domains, when compared to non-infected cells which show an increased number of smaller dots (**u**–**z**). Scale bar, 10 μm. Representative images of at least three independent experiments are shown; (**B**) Protein levels of SC-35, coilin and PML remain constant during ASFV infection. Vero cells infected with ASFV Ba71V isolate were lysed at 6 and 12 hpi, and compared to mock-infected cells, using immunoblotting analysis. α-Tubulin was used as loading control. Molecular weights (kDa) of evaluated proteins are indicated on the left of immunoblot images.

Some viruses commonly disrupt subnuclear domains that are involved in transcriptional events and in antiviral responses (reviewed in [35]). To unravel a putative crosstalk between ASFV and these domains, indirect immunofluorescence analysis of Vero-infected cells was performed from 4 h post infection (hpi) onwards, using antibodies that recognize the major constituent protein of the studied subnuclear domains (anti-SC-35 to identify nuclear speckles, anti-coilin to label Cajal bodies and anti-PML to detect PML-NBs), and an anti-ASFV swine whole serum to label infected cells. A disrupted morphology of these subnuclear domains was solely found in infected cells, from early times post infection (6 hpi). Nuclear speckles became reduced in number although enlarged in size (Figure 1A, a–d), as reported for other viral infections [36], and contrasting to non-infected cells (Figure 1A, e–h). Cajal bodies (CBs) were also altered from a dot distribution pattern observed in non-infected cells (Figure 1A, m–p), into “comma-shaped” structures and increased in number (Figure 1A, i–l). These rearrangements may be due to accumulation of snRNPs caused by the host transcriptional switch induced by ASFV [4]. Finally, PML-NBs were reduced in number and oversized in infected cells (Figure 1A, q–t), when compared to PML-NBs in non-infected cells (Figure 1A, u–z), similarly to the morphological changes observed in other DNA virus infections [37].

Considering that disruption of subnuclear domains may be related with an aberrant expression of its major constituents (SC-35, coilin and PML), their expression levels were evaluated by Western blot. Although, ASFV modulates their nucleoplasmic distribution, no differences were detected between non-infected and ASFV-infected cells (Figure 1B).

### 2.2. ATR-Related Factors Accumulate near PML-NBs during ASFV Infection

Recently, we have reported that ASFV specifically activates the Ataxia Telangiectasia mutated and Rad3-related (ATR) pathway, for a successful infection [6]. In non-infected cells, the ATR pathway is a homologous recombination (HR) signalling cascade, integrating different DNA damage response (DDR) mechanisms, which is p53-activation dependent and essential for the repair of stalled replication forks. Similarly, PML-NBs have been reported to display p53-dependent activities related to cell cycle control and to DNA damage sensing by HR mechanisms [38,39]. Indeed, PML-NBs and DDR factors have been found to cooperate in DNA repair after exposure to genotoxic agents [40,41].

**Figure 2:**
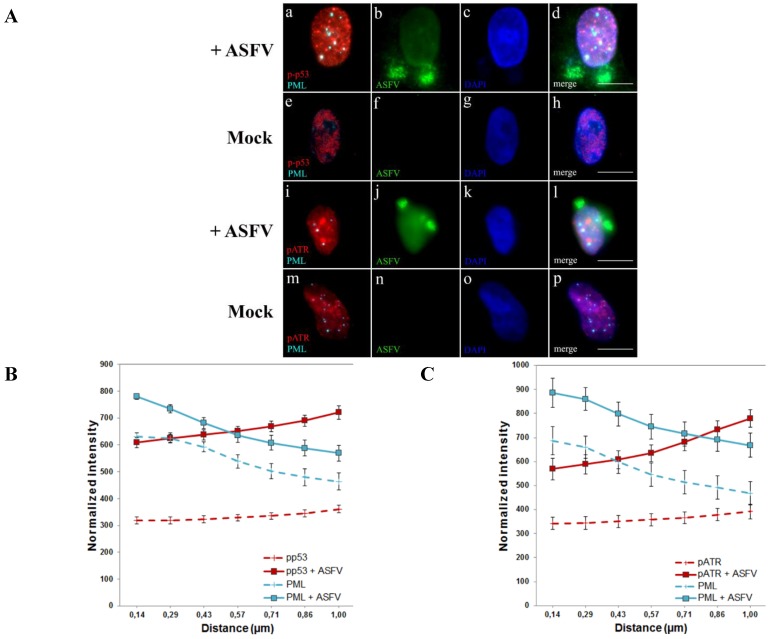
PML-NBs and DDR factors juxtapose during ASFV infection. (**A**) Immunofluorescence analysis of PML-NBs (cyan) and two DNA damage response factors (red) was performed in ASFV-infected cells (8 hpi, green) and in non-infected cells. Cell nuclei were counterstained with DAPI (blue). (**a**–**d**) In ASFV-infected cells, enlarged PML-NBs (cyan) are juxtaposed to phosphorylated p53 form (p-p53, red), while in non-infected cells these subnuclear domains do not associate to activated p53 loci (**e**–**h**). Additionally, enlarged PML-NBs neighbour pATR accumulations (**i**–**l**), as non-infected cells display smaller PML-NBs and pATR faint staining dispersed throughout the nucleus (**m**–**p**). Scale bar, 10 μm. Representative microscopy images of at least three independent experiments are shown; (**B**) and (**C**) Relative distance between PML-NBs and p-p53/pATR foci was evaluated by radial intensity profile analysis in ASFV-infected cells (solid lines) and non-infected cells (dashed lines). Normalized fluorescence intensity curves from the centre of PML-NBs (blue lines) to p-p53 accumulations (B) or to pATR foci (C) (represented by red lines) are plotted. Error bars represent standard errors (±SE). Radial profile analysis shows the close proximity between the subnuclear domains and the higher intensity DDR-factor accumulation regions only in ASFV-infected cells, as blue and red solid lines cross at a point of the studied radius, contrasting to the absence of intersection between PML-NBs (blue dashed lines) and p-p53/pATR fluorescence intensities (red dashed lines) in non-infected cells.

To investigate whether disrupted PML-NBs cooperate with DDR activated factors, distribution patterns of p-p53, pATR and γH2AX were analysed during infection. In ASFV-infected cells, PML-NBs became juxtaposed to p-p53 (Figure 2A, a–d), in comparison to non-infected cells (Figure 2A, e–h), and closely associated to pATR accumulations (Figure 2A, i–l), which was uncommon in non-infected cells (Figure 2A, m–p). Most probably, this close vicinity also occurs with γH2AX foci, although, because of its pan-nuclear distribution, this juxtaposition to PML-NBs could not be easily recognized in ASFV-infected cells (data not shown). The observed p-p53 and pATR accumulations corroborate the increased expression levels of ATR-related factors reported in ASFV infection [6], enhancing the proximity and functional crosstalk between activated DDR factors and PML-NBs thus interfering with the interferon-mediated antiviral responses, as described for other viral infections [40,42,43].

We analysed the redistributions quantitatively using a radial intensity profiling algorithm that measures the normalized integrated fluorescence intensity in immunostaining images of PML-NBs and DDR-related factors (p-p53 and pATR) from the subnuclear domains centre to a maximum radius distance of 1 µm (10 pixel). Radial profiles were obtained from 50 nuclei of ASFV-infected cells (solid lines) and 50 nuclei of non-infected cells (dashed lines) and average normalized integrated intensities were plotted as a function of distance. The results confirmed that the proximity between PML-NBs and pATR (Figure 2C), as well as the juxtaposition among PML-NBs and p-p53 foci (Figure 2B) occurs much more frequently in infected cells (solid lines), where the fluorescence intensity values of both PML-NBs (cyan lines) and DDR-related factors (red lines) are higher than in non-infected cells, meaning that ASFV infection orchestrates the nuclear distribution of PML-NBs and ATR-related factors.

### 2.3. PML Protein Plays a Proviral Role in ASFV Infection

Considering that PML-NBs are also recognized as transcriptional regulatory elements and post-translational modification platforms (reviewed in [19]), and become enlarged during ASFV infection, the role of PML protein on the viral infection progression was evaluated. For this purpose, we designed a shRNA to target the PML mRNA and the shRNA expression cassette (with a GFP reporter gene) was delivered via lentiviral transduction of Vero cells (Vero-shRNA-PML). This transduction system [44,45] was considered suitable for Vero cells due to an almost full sequence homology of PML gene between human and green monkey (DQ231470, EMBL—European Nucleotide Archive).

**Figure 3:**
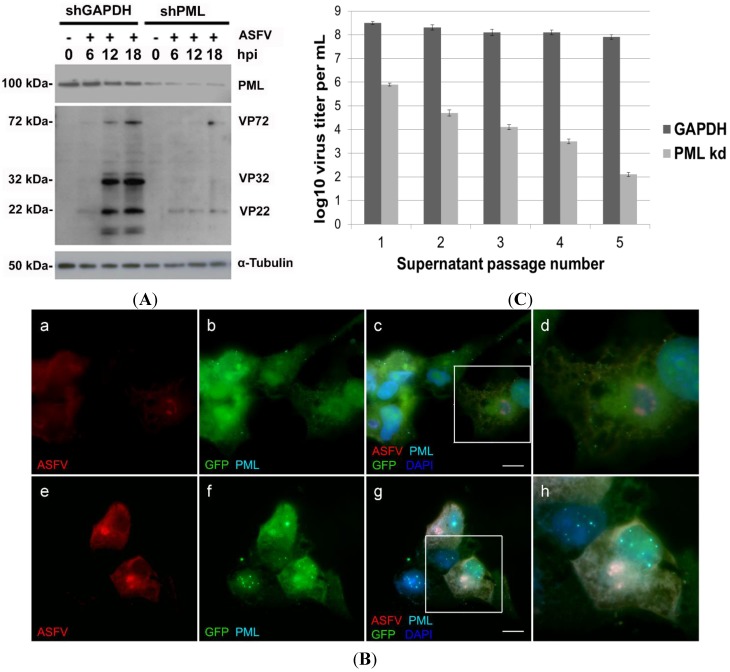
PML has a proviral role in ASFV infection. (**A**) Whole cell extracts were collected from Vero-shRNA-PML and Vero-shRNA-GAPDH cells, non-infected and infected with ASFV Ba71V isolate (MOI of 1) at 6, 12 and 18 hpi. In Vero-shRNA-PML cells, expression levels of a ≈22 kDa viral protein remain residual throughout infection, whereas in Vero-shRNA-GAPDH cells (control) this viral protein showed increasing levels. In addition, other structural viral proteins (≈32 kDa and ≈72 kDa) in ASFV-infected PML knockdown cells did not present the expression levels detected in infected shGAPDH cells, even at late times of infection. Overall, viral protein synthesis is diminished in Vero-shRNA-PML knockdown cells. As expected, mock-infected cells (0 hpi), showed no viral protein expression. α-Tubulin was used as loading control. Molecular weights (kDa) of evaluated proteins are indicated on the left of immunoblot images; (**B**) PML knockdown cells display lower intensity viral proteins staining and aberrant ASFV factories. (**a**–**d**) Vero-shRNA-PML cells (GFP expressing, green) were infected with ASFV (MOI of 1) and analysed at 12 hpi. PML (cyan) and ASFV-infected cells (red) were further detected by immunofluorescence. PML-NBs could not be visualized in PML knockdown cells, which show viral cytoplasmic factories with atypical morphology (horseshoe-shaped). (**e**–**h**) In contrast, Vero-shRNA-GAPDH cells (green) display enlarged PML-NBs and typical round-shaped viral factories (red). Scale bar, 10 µm. Representative images of at least three independent experiments are shown; (**C**) ASFV progeny is reduced in Vero-shRNA-PML kd cells. A drastic reduction in virus yields was observed in ASFV-infected PML knockdown cells (light grey columns) in comparison to the viral progeny production obtained from infected Vero-shRNA-GAPDH cells (dark grey columns). Each column represents the average of results obtained from three independent experiments, and the error bars represent the standard error (SE) values. Log decay of virus titer was considered as statistically significant (*p* value < 0.05).

The depletion efficiency was assessed by Western blot and, although highly efficient, a residual amount of native PML protein could still be detected in Vero-shRNA-PML cells. In contrast, transduction control cells (Vero-shRNA-GAPDH) showed high and constant expression levels of PML protein throughout ASFV infection (Figure 3A). Notably, low expression levels of viral proteins were detected in PML-knockdown cells, even at late times of infection (e.g., 12 hpi and 18 hpi), in contrast to the expected levels detected in infected Vero-shRNA-GAPDH cells (Figure 3A). As anticipated, no viral proteins were detected in mock-infected cells.

Immunofluorescence analysis of Vero-shRNA-PML cells revealed irregularly shaped viral factories (Figure 3B, a–d), again contrasting with Vero-shRNA-GAPDH cells that presented typical round-shaped factories and virus-induced PML-NBs disruption (Figure 3B, e–h). To further address whether ASFV infection is PML-dependent, viral yields obtained from Vero-shRNA-PML and Vero-shRNA-GAPDH cells were titrated. Initial ASFV Ba71V inoculum (MOI of 1) was adsorbed for 1 h and supernatants collected every 48 hpi were used for further passages, while a small aliquot was stored at −80 °C for subsequent viral titration [46]. Results showed that ASFV yields decrease six logs after five sequential passages in PML-knockdown cells, unlike the viral titers obtained from Vero-shRNA-GAPDH cells (*p* < 0.05). Titration results from three independent experiments are depicted graphically in Figure 3C.

### 2.4. ASFV Modifies Host Chromatin Epigenetic State

Recent studies have shown that some viruses subvert cellular epigenetic mechanisms and recruit host transcription factors to their benefit by changing chromatin structure (reviewed in [23]). In order to identify host chromatin rearrangements induced by ASFV, the distribution patterns of several epigenetic marks were analysed by immunofluorescence, using antibodies that specifically recognize different methylated forms of histone H3 (H3K9me1/me3, H3K27me1/me3 and H3K36me3), heterochromatin protein 1 isoforms (HP1α, HP1β, and HP1γ) and histone deacetylase enzymes (HDAC1, HDAC2, HDAC3, and HDAC5).

**Figure 4:**
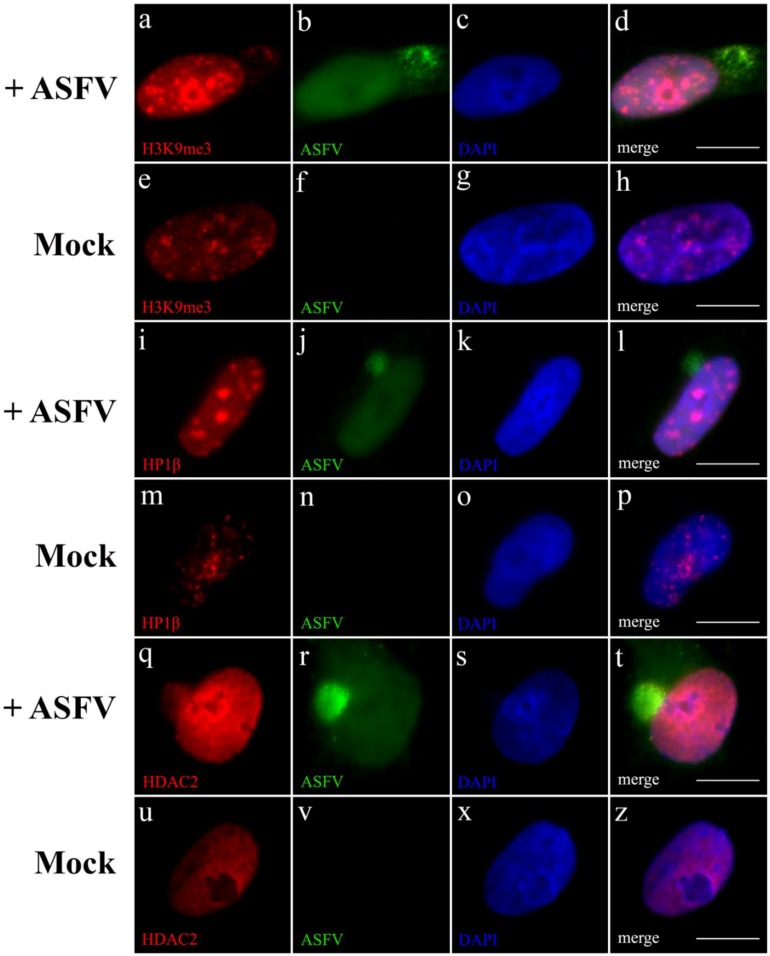
ASFV modifies the host chromatin state. Immunofluorescence analysis was performed on non-infected and ASFV-infected Vero cells (8 hpi) using specific antibodies recognizing heterochromatin marks—H3K9me3, HP1β and HDAC2 (red) and viral proteins (green). Cell nuclei were counterstained with DAPI (blue). (**a**–**d**) Histone H3 trimethylated at lysine 9 (H3K9me3, red) show large accumulations throughout the nucleoplasm upon ASFV infection (green), not observed in uninfected cells (**e**–**h**). ASFV-infected cells (green) also present HP1β (red) nucleoplasmic accumulations (**i**–**l**), not detected in non-infected cells (**m**–**p**). HDAC2 (red) is the only member of HDACs family that displays a more intense nuclear labelling in ASFV-infected cells (green), and additional recruitment to viral cytoplasmic factories (**q**–**t**), when compared to non-infected cells (**u**–**z**). Scale bar, 10 μm. Representative microscopy images of at least three independent experiments are shown.

As shown in Figure 4a–d, ASFV infection induces the redistribution of trimethylated lysine-9 of histone H3 (H3K9me3), an epigenetic mark specifically correlated with gene silencing in eukaryotic cells (reviewed in [47]), contrasting with its distribution pattern in non-infected cells (Figure 4e–h). A similar scenario was observed for trimethylated lysine-27 of histone H3 (H3K27me3), another epigenetic post-transcriptional modification involved in heterochromatin formation (data not shown). These virus-induced locations are characterized by few large accumulations located in the centre of the nucleoplasm. In contrast, no differences in the distribution patterns of several epigenetic marks associated to euchromatin formation (H3K9me1, H3K27me1 and H3K36me3) were observed (data not shown), all being used in the identification of transcriptionally active regions (reviewed in [48,49]).

Further, in non-infected eukaryotic cells, the balance between the different methylated forms of H3K9 regulates the recruitment of HP1 isoforms, which in turn cooperate with several chromatin-remodelling enzymes controlling gene transcriptional status (reviewed in [50]). Considering that these protein-protein interactions are major players in the regulation of host gene expression, intranuclear distributions of HP1α, HP1β and HP1γ were also studied during ASFV infection. Whereas HP1β displayed an accumulation pattern from early times of infection (Figure 4i–l), HP1α foci could only be detected after 10 hpi (data not shown) in contrast to the observed distribution patterns of both HP1 isoforms in non-infected cells (Figure 4m–p). These enlarged foci of both isoforms can solely be observed in ASFV-infected cells. In its turn, HP1γ isoform, which is mainly involved in euchromatin maintenance, does not relocalize in infected cells (data not shown).

Cellular chromatin texture and gene expression can also be regulated by histone deacetylases (HDACs) that modulate the histone acetylation status (reviewed in [51]). Knowing that H3K9me-HP1 interaction can recruit HDACs for heterochromatic regions [52], studies were carried out to verify HDACs localization in infected cells. Although all studied nuclear HDACs (class I—HDAC1, HDAC2 and HDAC3) partially localized with the cytoplasmic viral factories, only HDAC2 which regulates chromatin plasticity, revealed an increased signal intensity in ASFV-infected cells, from an early phase of infection (Figure 4q–t), not detected in non-infected cells (Figure 4u–z). Finally, no redistribution in the nucleocytoplasmic class II-HDAC5 could be detected in ASFV-infected cells (data not shown). Altogether, the above data strongly supports that ASFV promotes the heterochromatinization of the host nucleus by subverting different mechanisms, controlling the access of transcription machinery to host genes.

### 2.5. Subnuclear Domains and ATR Loci Accumulate nearby Heterochromatic Regions during ASFV Infection

**Figure 5:**
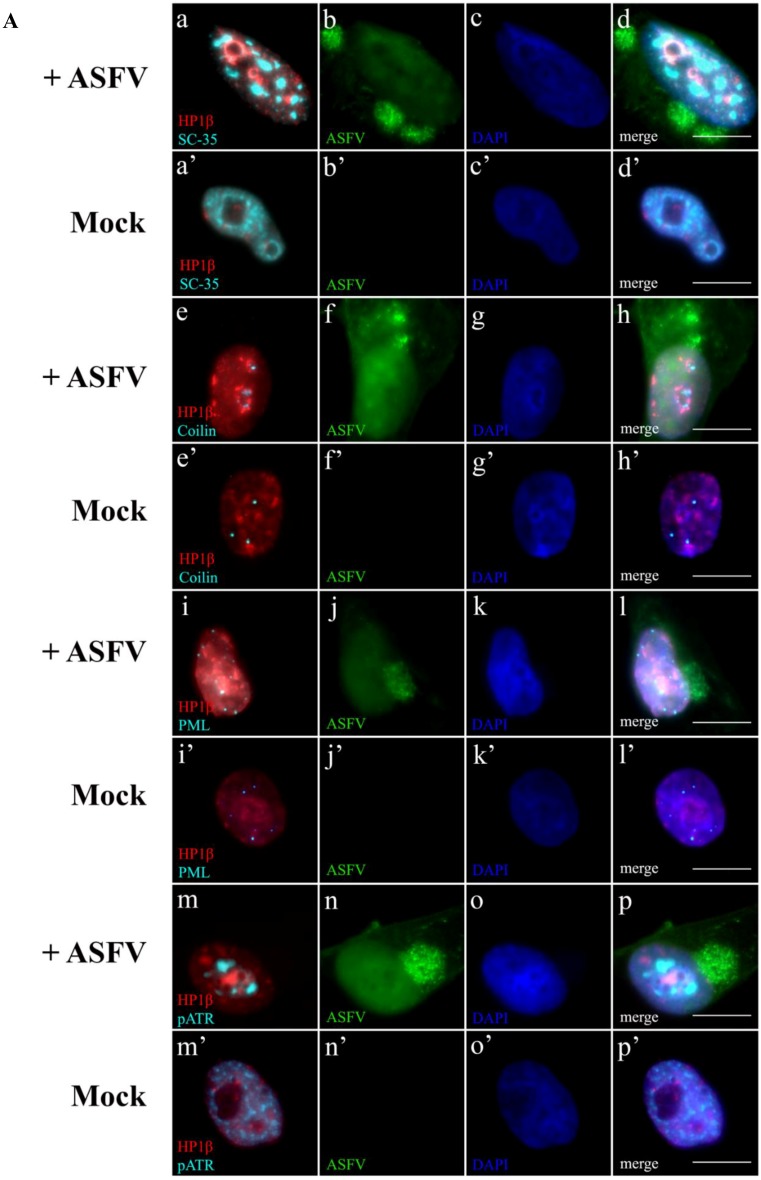

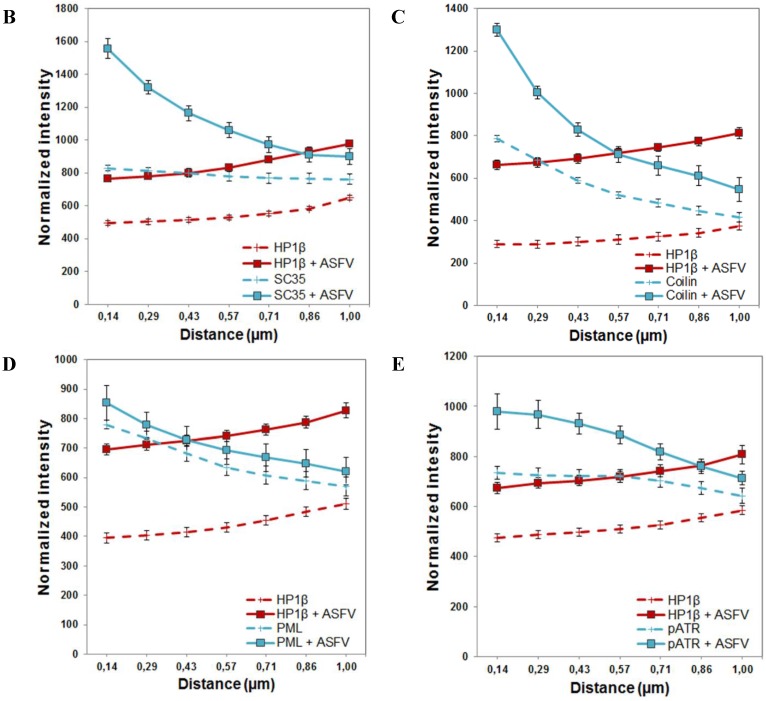
ASFV leads to juxtaposition of host heterochromatic regions, disrupted subnuclear domains and pATR foci. (**A**) Heterochromatin marker HP1β is labelled in red, while subnuclear domains (PML-NBs, nuclear speckles or Cajal bodies) and the activated ATR kinase are labelled in cyan. Cell nuclei were counterstained with DAPI (blue). (**a**–**d**) Only ASFV-infected cells (green) display the speckled pattern of enlarged SC-35 accumulations (cyan) juxtaposed to the heterochromatic regions (HP1 β, red), as non-infected cells do not reveal the close proximity pattern (**a’**–**d’**). In contrast to non-infected cells (**e’**–**h’**), the ASFV-induced bulky heterochromatic territories (HP1β, red) are always present within close vicinity to disrupted Cajal bodies (cyan) (**e**–**h**). Enlarged heterochromatic regions (HP1β, red) closely neighbour reorganized PML-NBs (cyan) in infected cells (green) (**i**–**l)**, rearrangements not observable in non-infected cells (**i'**–**l’**). HP1β deposits (red) juxtapose to pATR foci (cyan), in ASFV-infected cells (green) (**m**–**p**), different chromatin/pATR appearances and distributions in non-infected cells (m’–p’). Scale bar, 10 μm. Representative microscopy images of at least three independent experiments are shown; (**B**–**E**) Relative distance between the studied subnuclear domains/pATR foci (cyan) and heterochromatic regions (red) was evaluated by radial intensity profile analysis in ASFV-infected cells (solid lines) and non-infected cells (dashed lines). Normalized fluorescence curves were obtained from 50 nuclei. Experiments were performed in triplicate and error bars represent standard errors (±SE). Radial profile analysis shows the close proximity, in ASFV-infected cells, between each subnuclear domain/pATR foci (blue solid line) and HP1β accumulations (red solid line), crossing at a point within the given radius (1 µm). Dashed lines representing fluorescence intensities of subnuclear domains/pATR (blue) and heterochromatic regions (red) never intersect, revealing a greater distance between these nuclear domains/factors in non-infected cells.

In other viral infections, both subnuclear domains and DDR factors are recruited to heterochromatic loci, forcing the host transcriptional switch and DNA repair factors accessibility to enhance viral replication (reviewed in [53]). This knowledge led us to investigate if juxtaposition occurs between heterochromatic host regions, subnuclear domains and ATR accumulations during ASFV infection. Our results show that disrupted nuclear speckles and Cajal bodies became juxtaposed to hyper-condensed chromatin aggregations (identified by HP1β accumulations), only in ASFV-infected cells, (Figure 5a–h, respectively), contrasting with the patterns observed in non-infected cells (Figure 5a’–h’, respectively). These viral-induced HP1β foci also displayed close proximity to PML-NBs (Figure 5i–l), showing different distribution patterns when compared to non-infected cells (Figure 5i’–l’), and close vicinity to pATR accumulations (Figure 5m–p), in opposition to the diffuse distribution pattern found in non-infected cells (Figure 5m’–p’). The radial-analysis method was further applied to subnuclear domains, DDR-related factors accumulations and to heterochromatic regions providing consistent results of differential intensity profiles when infected and non-infected cells were compared (Figure 5B–E). At the given distance of 10 pixel (1.0 µm), average normalized integrated fluorescence profiles of nuclear speckles (Figure 5B, cyan lines), Cajal bodies (Figure 5C, cyan lines), PML-NBs (Figure 5D, cyan lines) and pATR accumulations (Figure 5E, cyan lines) showed closer proximity to heterochromatic regions (HP1β labelling, red lines) which presented much higher fluorescent intensity values in ASFV-infected cells (solid lines) than in non-infected cells (dashed lines).

## 3. Discussion

It is known that different viruses exploit subnuclear domains to thrive a successful infection [13,17]. Our results demonstrate that ASFV also disrupts nuclear speckles and Cajal bodies morphology, probably to favour a host transcriptional switch (reviewed in [4]), and to induce downregulation of host splicing events. Although the role of PML-NBs in ASFV infection remains to be fully understood, their remodelling herein described share similar features to those reported in other viral infections, where these modified domains provide the required physical architecture for an efficient synthesis of viral proteins and/or constitute protein deposits that can assist viral replication (reviewed in [54,55]). Indeed, some DNA viruses (e.g., Papillomaviruses and Polyomaviruses) localize their replication centres within or at the periphery of PML-NBs to accomplish their infection cycle (reviewed in [56,57]), while Herpesviruses have been found to block PML-NBs antiviral responses by the mislocation of these domains [58]. Our findings from PML knockdown experiments strongly support that ASFV infection is PML-dependent, since its depletion leads to irregularly shaped viral factories and to a significant reduction in viral progeny release. However, and despite the spatial reorganization of the above-mentioned subnuclear domains, the protein levels of their major constituents (SC-35, coilin and PML, respectively) remained unchanged throughout infection.

In eukaryotic cells, PML protein has also been implicated in DNA repair responses by acting as sensor and regulator of p53 [39,59], both functions corrupted by several viruses to circumvent cellular antiviral measures [11,60,61]. Here, we show that disrupted PML-NBs share a close vicinity to p-p53 and pATR accumulations, suggesting that ASFV may exploit this scenario to increment the recognition of its genomes, corroborating the previously identified ATR pathway activation [6].

Meanwhile, the host chromatin also represents a challenge to the establishment of viral infections. By controlling histone methylation/acetylation status and distribution of HP1 isoforms, viruses modify the chromatin texture, promoting a permissive nuclear environment and subverting host gene expression (reviewed in [9,23,53]). In eukaryotic cells, active genes are mainly associated to euchromatin, whereas silenced heterochromatic regions are H3K9me3-enriched and tend to replicate later [29,48]. It is well-known that H3K9me3 is involved in the recruitment of major heterochromatin-related proteins (HP1α and HP1β), which in their turn reinforce and stabilize heterochromatic regions, contrasting with HP1γ isoform that typically accumulates in euchromatin (reviewed in [62]). Further, the precise intranuclear distribution of HP1 isoforms contribute to the fine-tuning of DNA damage signalling and to chromatin repair by HR mechanisms, events exploited by some viruses to inhibit expression of cellular harmful genes and to take advantage of the DNA repair mechanisms for replicating their genomes [20,24,26,53,63]. The results here reported link ASFV infection with the redistribution of heterochromatin-related marks (H3K9me3, H3K27me3, HP1α, HP1β and HDAC2), while distribution patterns of euchromatin marks (H3K9me1, H3K27me1, H3K36me3 and HP1γ) remained unchanged. These virus-induced epigenetic modifications reflect the heterochromatinization of the host nucleus and probably occur to silence specific host gene clusters that encode harmful proteins to the virus (e.g., apoptotic and IFN responses). Additionally, HP1α and HP1β redistributions observed in ASFV-infected cells can explain the nuclear lamina breakdown reported during ASFV infection [5], since both HP1 isoforms are known to be involved in the nuclear envelope assembly [28]. Moreover, considering that ASFV has an intranuclear DNA replication phase [8], the disintegration of the nuclear lamina may also be related to structural changes induced by viral replication compartments and/or by viral nuclear entry/egress, as reported in other viral infections [64,65,66,67]. Even thought, the observed intranuclear changes could result from the nuclear lamina disassembly, the detected redistribution of heterochromatin-related factors are time and space-dependent in infected cells, while euchromatin-related factors and most of the HDAC enzymes did not show altered distribution patterns, thus favouring a non-stochastic event and not only linked to the nuclear envelope breakdown. Presently, HDACs are recognized as partners of different replication complexes, for their ability to remove acetyl groups at specific histone tail residues and for inducing heterochromatin formation [28,51], and recent data on Herpesvirus infection suggest that molecular interactions between H3K9me3 and HP1 proteins recruit HDACs to viral factories to enhance viral replication [33]. Our data reveal that HDAC1, HDAC2, and HDAC3 are recruited to ASFV cytoplasmic factories, with special emphasis for HDAC2, that is related to chromatin plasticity [68]. This viral-induced redistribution may either interfere with the host epigenetic status or favour ASFV replication as reported for other viral infections [69]. Even though the role of HDAC6 in ASFV cytoplasmic factories formation is still to be proven [70], our findings on class I HDACs raise the possibility of using HDAC I inhibitors to disrupt ASFV infection, as demonstrated in other viral infections [71,72].

**Figure 6:**
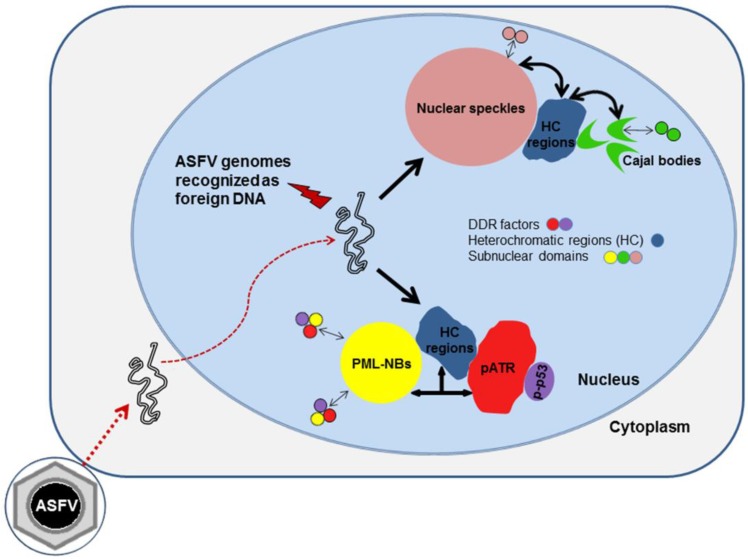
Proposed working model for ASFV-host interactions. ASFV-infected cells show a fully reorganized nuclear architecture. ASFV genomes most probably recognized as DNA damage sites, after migrating into host cell nucleus, activate DNA damage response factors (p-p53 and pATR), that juxtapose to enlarged PML-NBs (yellow circles). The activated p53 (p-p53, purple circles) and ATR (pATR, red shapes) also accumulate nearby heterochromatic regions (blue forms). In addition, the viral infection promotes nuclear speckles enlargement (pink circles) and Cajal bodies remodelling (green “comma-shaped” forms). All subnuclear domains display close vicinity to viral-induced heterochromatic regions enriched by H3K9me3, H3K27me3, HP1α/β isoforms and HDAC2.

Taken together, our results show that ASFV disrupts subnuclear domains and modulates several chromatin-related mechanisms in order to possibly create a repressive nuclear environment. This nuclear landscape is further strengthened by the recruitment of the subnuclear domains to heterochromatic regions, emphasizing the ASFV-induced host transcriptional switch, as also described for Herpesvirus [10,73]. Finally, the radial profile analysis showed the spatial proximity of subnuclear domains, heterochromatic regions and DDR-related factors in ASFV-infected cells. These findings are schematically illustrated in the proposed working model for ASFV-host interactions presented as Figure 6. When ASFV enters the host cell, viral genomic material is detected as a foreign DNA molecule promoting the juxtaposition of pATR, p-p53 and PML-NBs. By changing the distribution patterns of several epigenetic marks and HDACs, ASFV induces nuclear heterochromatinization probably to silence harmful genes and to increase interchromatin space, settling extra room for its intranuclear replication events. Furthermore, the proximity between the disrupted subnuclear domains and the heterochromatic regions may facilitate the sequestration of host factors and their recruitment to viral replication sites.

In sum, the novel host-virus interactions herein described, disclose unknown molecular mechanisms that are subverted by ASFV, emphasizing the role of the host nucleus for the establishment of a productive infection. This knowledge provides information that supports several inhibitors to be tested as potential anti-viral drugs (e.g., PML, HP1 and HDAC I inhibitors), and opens new insights for the identification of viral genes involved in the reported viral-host interactions towards the development of improved vaccines against ASFV.

## 4. Materials and Methods

### 4.1. Vero Cell Culture and Lentiviral Infection of shRNA

Vero cells (kidney epithelial cells of African green monkey *Cholorocebus aethiops*) were obtained from the European Cell Culture Collection (ECACC, Salisbury, UK), and maintained in DMEM (Dulbecco Modified Eagle's minimal essential medium) supplemented with L-Glutamax, 10% foetal calf inactivated serum and non-essential amino acids (all from Gibco, Life Technology, Karlsruhe, Germany). All cell culture experiments were conducted in the absence of antibiotics or antifungal agents and were performed on actively replicating subconfluent cell monolayers.

In order to knockdown PML protein, Vero cells were seeded into 24-well plates (1.5 × 10^4^ cells/well) and infected with pGIPZ-shRNA-PML fused to TurboGFP lentiviral particles (Thermo Scientific, Waltham, USA), at the recommended multiplicity of infection (MOI) of 1, as previously reported [74,75]. Vero cells transduced with pGIPZ-shRNA-GAPDH replication incompetent lentivirus were used as a transduction control. After 6 h of lentivirus adsorption at 37 °C, cells were incubated with supplemented DMEM. In both transduced cell populations, GFP expression, from pGIPZ-TurboGFP vector, allowed the lentiviral infection efficiency measurement and further selection of transduced cells. Transduction protocol optimization was performed according to the manufacturer’s recommendations and the selective pressure was performed by adding puromycin (5 µg/mL; Sigma-Aldrich, St. Louis, MO, USA) for 48 h after lentiviral infection. After two selecting periods, the GFP-expressing cells were screened through observation using an inverted epifluorescent microscope (Olympus LH 50A, Tokyo, Japan) and named as Vero-shRNA-PML and Vero-shRNA-GAPDH. Selective pressure was maintained for 10 days and stable knockdown cells were confirmed by puromycin resistance and GFP-expression. All cell cultures were grown at 37 °C, under a 5% CO_2_ and humidified atmosphere (≥95%).

### 4.2. Virus and Infections

The ASFV Ba71V isolate was propagated and prepared as previously described [46]. Viral suspensions titrations were performed by observation of cytopathic effect (CPE) at end-point dilutions, in Vero cells, as previously described [76]. Infections were carried out with a MOI of 2 for Western blot analysis and a MOI of 5 for immunofluorescence studies. PML-knockdown cells (Vero-shRNA-PML) and transduced controls (Vero-shRNA-GAPDH) were infected using a MOI of 1. For evaluation of viral yields, supernatants of infected cultures were diluted in serum-free medium, adsorbed to cell layers and incubated for 1 h. After this adsorption period, supernatants were collected and replaced by fresh culture medium.

### 4.3. Antibodies

Subnuclear domains were detected with the following antibodies: goat anti-SC35 (sc-10252; IIF 1:50; WB 1:500), rabbit anti-coilin (sc-32860; IIF 1:50; WB 1:500) both from Santa Cruz Biotechnology, Santa Cruz, USA; and, rabbit anti-PML (ab96051 from Abcam, Cambridge, UK; IIF 1:100; WB 1:1000). A rabbit anti-αTubulin was used for immunoblotting loading control (#2125, Cell Signaling Technology, Boston, MA, USA; 1:1000). Activated DDR factors were detected by antibodies that specifically recognize p-p53 (#9284, 1:50) and pATR (#2853, 1:100), both from Cell Signaling.

Chromatin marks immunostaining was performed with: rabbit anti-HP1α (#2616; 1:100), rabbit anti-HP1β (#8676; 1:100), rabbit anti-HP1ɣ (#2619; 1:100), mouse anti-HDAC1 (#5356; 1:400), mouse anti-HDAC2 (#5113; 1:800), mouse anti-HDAC3 (#3949; 1:100), rabbit anti-HDAC5 (#2082; 1:100) all from Cell Signaling (Boston, USA). The histone H3 methylation status was characterized by: rabbit anti-H3K9me1, rabbit anti-H3K9me3, rabbit anti-H3K27me1, rabbit anti-H3K27me3 and rabbit anti-H3K36me3 antibodies, kindly provided by Professor Thomas Jenuwein (Max Planck Institute of Immunobiology and Epigenetics, Freiburg, Germany) and used as previously reviewed [47]. For viral protein detection, an in-house produced swine anti-ASFV serum was used (IIF 1:100, WB 1:500).

The secondary fluorescent-conjugated antibodies were used as follows: anti-mouse FITC (sc-2099, Santa Cruz Biotechnology; 1:500); anti-mouse Cy3 (A10521, Molecular Probes, Life Technologies, Paisley, UK; 1:900), anti-rabbit DyLight 594 (ab98490, Abcam; 1:500), anti-swine Texas Red or FITC (ab6775 and ab6773, respectively, Abcam; 1:500).

For Western blot analysis, primary antibodies were revealed with horseradish peroxidase-conjugated secondary antibodies (Jackson ImmunoResearch Lab., West Grove, PA, USA), at optimized concentrations during a 30 min incubation period.

### 4.4. Immunofluorescence Studies

Vero cells were seeded and grown onto glass coverslips, in 24-well culture plates (5.0 × 10^4^ cells/cm^2^) and infected with ASFV Ba71V isolate. At different time points, cells were fixed with 3.7% paraformaldehyde (PFA) in HPEM buffer (25 mM HEPES, 60 mM PIPES, 10 mM EGTA, 1 mM MgCl2), for 10 min at room temperature (RT) and further permeabilized in PBS/Triton X-100 0.1% for 2 min. Bovine serum albumin (5% in PBS/Tx-100 0.005%) was used during 30 min, at RT, as a blocking step prior to incubation with primary antibodies. All antibodies were diluted in blocking solution and incubated at the recommended periods. DAPI (4,6-diamidino-2-phenylindole) was used to stain DNA, and Prolong Gold antifade (Invitrogen, Carlsbad, CA, USA) was used as mounting medium. Special attention was taken to prevent fluorochrome fading by using a dark humidified chamber in all incubation steps.

### 4.5. Microscopy and Image Processing

Images were acquired on a Leica DMRA2 upright microscope (Leica Microsystems, Wetzlar, Germany) equipped with a CoolSNAP HQ CCD camera (Photometrics, Tucson, AZ, USA), using the 100× 1.4NA Oil immersion objective, DAPI, FITC, FM 4-64 and CY5 fluorescence filter sets. MetaMorph software (version 7.5.3.0, Molecular Devices, Sunnyvale, CA, USA) was used to collect stacks of images with a z optical spacing of 0.2 μm. Data sets preparation and analysis was performed within the open-source Java-based image processing program FIJI, version 1.47q [77], which was also used to generate maximum intensity projection images along the z axis.

The radial intensity analysis of images was based on a previously described method [78] and performed using an in-house developed macro that used the Radial Profile Extended plug-in for ImageJ. In brief, radial profile measurements were centred on each subnuclear domain/HC region/DDR factor loci, defined by specific signals. Region of interest radii were set to extend from the local centre of these subnuclear dense structures to its boundary, set at 10 pixels, and integrated intensity data (sum of pixel intensity values at a given radius) was normalized by dividing the number of pixels in the corresponding radius. Nuclei of 50 non-infected and 50 ASFV-infected cells were used for each analysis. Control sites were selected randomly in the corresponding nuclei.

### 4.6. Radial Analysis of Site Images

Kruskal Wallis and Mann-Whitney tests for median comparison were performed to analyse the significance of the observed subnuclear domains enrichments between ASFV-infected and non-infected cells, using IBM SPSS Statistics version 21.0 software (IBM Corp. New York, NY, USA). Data are presented as mean ± SE (50 non-infected and 50 ASFV-infected cells were analysed for each condition). Differences with *p* values of less than 0.05 were considered to be statistically significant.

### 4.7. Western Blotting Analysis

ASFV Ba71V infected or mock infected Vero cells and Vero-shRNA transduced cells were harvested at indicated times. After being washed with phosphate-buffered saline (PBS), cells were lysed with ice-cold RIPA buffer (50 mM Tris-HCl, pH 8.0; 150 mM NaCl; 1.0% NP-40; 0.5% sodium deoxycholate; 0.1% SDS) supplemented with protease and phosphatase inhibitors (cOmplete Mini, EDTA-free and PhoStop both from Roche, Mannheim, Germany). Clarified whole-cell extracts were resolved by SDS-polyacrylamide gel electrophoresis and electroblotted onto nitrocellulose membrane with a 0.2 µm pore diameter (Whatman, Dassel, Germany). Thereafter, membranes were blocked with TBS plus 0.05% Tween 20 (TBST buffer) containing 1% BSA, during a 30 min incubation period at RT. Blots were incubated with the specific primary antibodies during 1 h, subjected to three 10-min wash steps with TBST buffer and then exposed to horseradish peroxidase-conjugated secondary antibodies (30 min, RT), followed by chemiluminescence detection through autoradiography (Clarity, ECL detection system, Bio-Rad Laboratories, Hercules, CA, USA), on Amersham hyperfilms (GE Healthcare, Little Chalfont, UK).
